# Exploring potential barriers and facilitators to integrate tuberculosis, diabetes mellitus, and tobacco control programmes in India

**DOI:** 10.7189/jogh.15.04230

**Published:** 2025-08-15

**Authors:** Nisha Mutalikdesai, Kajal Tonde, Kanchan Shinde, Rakesh Kumar, Surbhi Gupta, Girish Dayma, Anand Krishnan, Sanjay Juvekar, Ailana Santosa, Nawi Ng, Rutuja Patil

**Affiliations:** 1 Community Health Research Unit, KEM Hospital Research Centre, Pune, India; 2 Vadu Rural Health Program, KEM Hospital Research Centre, Pune, India; 3 Centre for Community Medicine, All India Institute of Medical Sciences, New Delhi, India; 4 Institution of Medicine, School of Public Health and Community Medicine, Sahlgrenska Academy, University of Gothenburg, Gothenburg, Sweden

## Abstract

**Background:**

Co-integrating tuberculosis (TB), diabetes mellitus (DM), and tobacco control (TC) programmes in India could help address the triple burden of these diseases. However, limited information exists regarding the feasibility and determining factors of such integration. We explored potential barriers and facilitators to integrating TB, DM, and TC programmes in Ambegaon Block of Pune District, Maharashtra, and Ballabgarh Block of Faridabad District, Haryana, in India.

**Methods:**

We conducted a qualitative study based on in-depth interviews with health workers, programme managers, and stakeholders involved in TB, DM, and TC programme implementation whom we enrolled using purposive and snowball sampling. The interview guide was based on World Health Organization's Health System Strengthening framework. We collected the data between November 2022 and March 2023 and analysed it through the rapid analysis method.

**Results:**

We interviewed 32 participants. The major challenge for integration, according to the participants’ perspectives, relates to the level of service delivery, which is primarily attributed to inadequate implementation of all the programmes. Themes that emerged as facilitators were well-designed programmes with robust guidelines and ample space for infrastructure, while those seen as barriers included inadequate referral systems, insufficient infrastructure, limited resources, a shortage of trained staff, and a lack of essential drugs and equipment, all of which impeded the uptake and coverage of services.

**Conclusions:**

Our findings highlight the critical importance of addressing barriers and facilitators to implementing programmes in India for tackling the triple burden of TB, DM, and TC. A multidimensional approach and targeted strategies are needed for overcoming these challenges. Sensitising the health system staff, implementing feedback and referral systems, and developing cross-programme digital platforms will offer a roadmap for policymakers and healthcare system managers.

According to the World Health Organization (WHO), India accounted for more than two-thirds of the global tuberculosis (TB) cases in 2020, corresponding to the highest burden of TB worldwide and the second-highest burden of diabetes mellitus (DM) [1]. According to Global Burden of Diseases data for 2019, TB is still one of the top ten causes of disability-adjusted life years, despite a recent rapid epidemiological change [2]. The triple burden of TB, DM, and tobacco addiction poses a threat to many TB-endemic countries that have not attempted to address them concurrently [3], demanding a shift from the traditional TB-centric approach to this issue. Considering the increased global risk posed by infectious diseases and non-communicable diseases (NCDs) comorbidity, a collaborative preventive approach can be employed to alleviate the strain on overburdened healthcare system. Specifically, an approach integrating TB, DM, and tobacco control (TC) programmes may be considered effective in detecting and treating TB cases, as well as mitigating the residual burden of intersecting factors that contribute to a high annual incidence of TB morbidity and mortality.

Given the prevalence of intersecting TB-DM epidemics, many countries have adopted the WHO collaborative framework [4] for developing preventive TB-DM co-management programmes. India also launched such an initiative through the National Tuberculosis Elimination Program (NTEP), but failed to generate sufficient evidence on health system readiness and obstacles associated with the framework's adoption [5].

The NTEP, formerly known as the Revised National TB Control Programme, is one of the biggest health programmes in the world. Initially launched in 2010, India has implemented the National Programme for Prevention and Control of Cancer, Diabetes, Cardiovascular Diseases, and Stroke (NPCDCS) in March 2016, which is now known as the National Programme for Prevention & Control of Non-Communicable Diseases. In line with the global framework established by the WHO and the International Union Against Tuberculosis and Lung Disease in 2011, NTEP and NPCDCS developed a joint framework in 2017. The Government of India established the National Tobacco Control Program (NTCP) in 2007–08 as part of the 11th Five-Year Plan.

Eliminating TB by 2025 remains an ambitious, yet imperative goal, considering the challenges posed by sociocultural practices and the country’s healthcare system [5]. The lack of awareness among healthcare practitioners and the public regarding the complex relationship between TB and DM results in missed opportunities for early identification [6]. Shared symptoms of these conditions, such as fatigue, weight loss, and increased susceptibility to infections, further compound diagnostic complexities, delaying essential care. By building upon the findings of previous studies [1,7,8], we highlight these challenges within the specific context of TB and DM integration, offering novel insights into the unique hurdles faced in achieving a comprehensive approach to healthcare.

Given that DM elevates the risk of TB and tobacco worsens the adverse effects of both TB and DM, any healthcare programmes should attempt to address all three factors simultaneously. The existing fragmented approach in India yields poor health outcomes and higher healthcare costs by overlooking opportunities for early diagnosis and efficient treatment. Through early detection and thorough risk factor management, integration can improve overall patient management, maximise resources, and improve health outcomes. By ensuring coordinated care, ongoing monitoring, and minimising total healthcare costs, this integrated method is anticipated to reduce disability-adjusted life years and prevent fatalities significantly. Here, we focussed on the integration of TB and DM programmes, exploring critical challenges that hinder timely detection and effective management.

Considering a lack of evidence on the incorporation of tobacco control initiatives, we aimed to explore the facilitators and barriers associated with the seamless integration of TB-DM-TC activities within India’s healthcare landscape.

## METHODS

### Study design and setting

In this qualitative, interview-based study, we aimed to identify potential barriers and facilitators to integrating TB, DM, and TC programmes in India, as seen by a diverse range of stakeholders. We conducted the study in two Indian districts: one in the state of Maharashtra and the other in Haryana, between November 2022 and March 2023. The interviews themselves took place in various settings, including health facilities such as clinics, primary health centres, state health system resource centres, and the programme implementation government cell in rural regions of both states.

### Participants and recruitment

We used purposive and snowball sampling to recruit participants with a diverse range of experiences and perspectives. We first contacted them *via* email or phone to inform them about the study’s purpose and objectives, and to confirm the location and time of the interview based on their willingness to participate, their availability, and feasibility. Those who consented to participate in in-depth interviews (IDIs) were given an information sheet and a consent form which they had to read and sign prior to enrollment.

### Data collection

NM, KT, and KS conducted the study IDIs in Maharashtra, while SG and RK conducted those in Haryana. Most interviewers were female, with backgrounds in public health and qualitative research, particularly in health systems and chronic disease management. They had no prior relationships with the participants before the start of the study.

The data collection and analysis were guided by the WHO’s Health System Strengthening (HSS) framework [9], which outlines six building blocks for health system analysis and improvement. The IDI guide therefore comprised six sections: service delivery, health workforce, health information system, financing, governance and leadership, and access to essential medicine, which investigated current service provision strategies for TB and DM care. These sections were used to explore the barriers and facilitators influencing the delivery of TB, DM, and TC services, and to assess the feasibility of implementing an integrated programme addressing all three healthcare challenges (File S1 in the **Online Supplementary Document**).

The IDIs, which lasted 45 minutes on average, were conducted face-to-face and audio-recorded, with a trained note-taker taking notes. We did not conduct repeat interviews.

### Data analysis

We employed the rapid analysis approach to code the data [10]. The study team members (NM, KT, KS, SG, and RK) who collected the data listened to the audio-recorded interviews and coded relevant excerpts using a rapid analysis matrix created in in Excel sheets following a deductive approach, whereby initial codes and the sub-codes were derived from the data collection tools., *i.e.* the WHO HSS framework [9]. During the coding process, the team iteratively refined the coding framework in line with WHO HSS framework by incorporating additional inductive codes that emerged from the data. They later translated the rapid analysis matrix from Marathi/Hindi (*i.e. *local languages) into English. Finally, they mapped the summarised data to the corresponding WHO HSS building blocks for further analysis and interpretation.

## RESULTS

### Participants characteristics

We conducted a total of 32 IDIs, with 13 participants being women and 19 being men. One participant was a national-level stakeholder (from NTEP), 11 were state-level stakeholders (seven from NTEP, two from NPCDCS, and two from NTCP), 17 were district-level stakeholders (accredited social health activist workers, medical officers, community health officers, laboratory technicians, *etc.*, with direct involvement in the fieldwork for all the programmes), one was a researcher (from NPCDCS), and two were representatives of non-governmental organisation (from NTEP).

**Figure 1:**
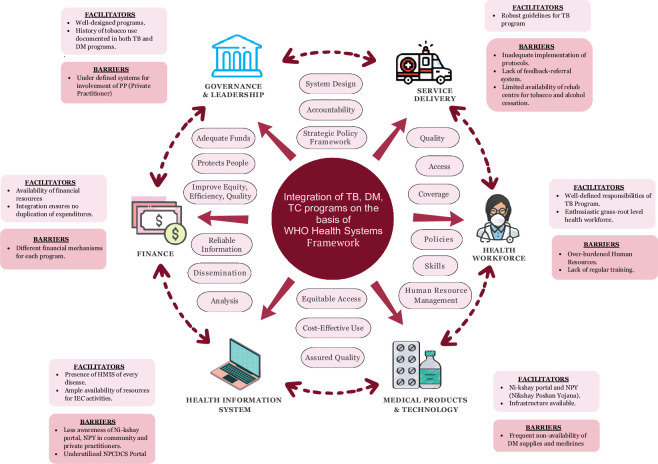
Illustration of the connection between each building block and essential findings.

We organised the study's findings according to the WHO HSS framework building blocks, with each team arranged under each block (**Figure 1**).

### Service delivery

Service delivery refers to the process by which healthcare services are delivered and managed. The Indian health system's current service delivery structure and regulations act as both facilitators and barriers.

#### Inadequate implementation of protocols

While the data confirm the existence of TB-DM and TB-TC frameworks, a lack of detailed operational guidelines has led to inadequate collaborative efforts across these programmes. While DM screenings are conducted for all TB patients, not all DM patients exhibiting the four-symptom complex (*i.e.* cough of any duration, fever, night sweats, weight loss) undergo screening for TB. This deficiency in service delivery contributes to a neglect of symptoms and a reduced motivation to visit health facilities. Inadequate monitoring, inactive management surveillance, and infrequent public awareness campaigns further hinder the effectiveness of bi-directional TB-DM screening and smoking control initiatives.

If we see the TB report, 90% of the TB patients are screened for diabetes. There are systems and mechanisms for that. Nik-shay is there; screening and monitoring is happening meticulously. But if we look at it the other way, there is a gap in the implementation for screening of TB in NCD clinics. Even though the framework is present, there are no operational guidelines as such.* – IDI# 22*

#### Lack of linkage mechanisms

The integration of the TB-DM-TC programme faces barriers due to an inadequate referral system, hindering proper management and continuity of care for patients with co-infections. The absence of communication between primary care facilities and referral hospitals undermines progress and potential enhancements.

(…) referral and feedback mechanisms are not strong at all. Testing is happening in NCDs, but we don't get any feedback from the TB patients that are sent here in these NCD clinics. *– IDI# 17*

Participants highlighted the absence of links in the referral system, indicating potential disruptions in patient follow-up processes. Issues they noted in rural healthcare delivery encompass non-compliance with the system, self-referrals to doctors, and direct referrals to specialists.

#### Limited availability of TC centres

The participants identified that the limited availability of TC centres hinders the integration of TC services into the TB-DM programme. Inadequate infrastructure, resources, trained staff, and essential drugs and equipment impeded the uptake and coverage of services. Consequently, there is low awareness and insufficient support for individuals struggling with tobacco addiction.

For tobacco control and counselling, there is no provision for below taluka (block) level, everything happens till block level. Dedicated centres are there along with dentists and counsellors, but not all are in a working condition. – *IDI# 9*

The insufficient presence of trained healthcare professionals further restricted the implementation of the NTCP initiative and contributed to accessibility challenges. Limited awareness regarding the detrimental effects of tobacco has led to a scarcity of TC centres, resulting in a low demand for services and reluctance among individuals to seek access.

#### Relatively robust programme guidelines for TB when compared to DM

All participants unanimously agreed that decentralising the effective TB control programme down to the primary healthcare and private health facility level serves as an appropriate starting point for the integration of TB-DM-TC programmes. They indicated that the existing TB programme guidelines were firmly established and widely implemented within the Indian health system. These guidelines laid a robust foundation and framework for integrating DM and smoking control services.

NTEP is a well-structured programme with good human resources. TB services are provided free of cost. There is a Ni-shay portal for reporting the patients. The programme has strong guidelines and protocols in place. The NCD programme is a little weak. Very poor mechanism of referring to the patients. *– IDI# 2*

The Indian health system lacks well-developed guidelines for DM, with patients managed within general services without a specialised section. This probably leads to abrupt medical attention and assistance to DM patients, creating difficulties in accessing necessary care, even in emergencies.

### Health workforce

The health workforce theme addresses the availability, responsibilities, training, and remuneration of healthcare personnel in integrated care, as well as the barriers and facilitators to delivering integrated care.

#### Well-defined responsibilities for the implementation of TB programme

While the NTEP in India has designated a workforce responsible for different levels of implementation, the DM programme, conversely, receives minimal attention with no designated staff. The roles and responsibilities within the DM programme are unclear. The implementing staff at the public healthcare facilities have limited understanding of programme guidelines, leading to ambiguity and confusion that hinder effective implementation and the integration of TB and DM screening.

Because of the lack of attention paid to diabetes care, there is no reference guideline. As a result, we have been diagnosing and managing diabetes patients in a haphazard way. Also, the NPCDCS programme has many diseases clubbed together because of which there is no separate staff present only for DM. *– IDI# 12*

#### Overburdened human resources

Healthcare workers in the study areas face overwhelming workloads in implementing routine programmes, reporting, procurement, and overseeing critical segments. The current health workforce is insufficient to meet service demands and embrace new initiatives. Introducing integrated TB and DM screening programmes might amplify workloads, potentially leading to burnout, fatigue, and stress, and causing a detrimental effect on productivity and efficiency.

There is a lot of workloads on us. We do not even get proper incentives for the amount of work we are doing. We have to keep a check on all the people in our area for all diseases. Apart from our work, sometimes we have to manage primary health care work also because there are less people working here. *– IDI# 14*

#### Lack of regular training for the healthcare personnel

Ensuring regular training for healthcare personnel is crucial for managing TB and DM cases. However, resource limitations and competing priorities often hinder training opportunities, resulting in delayed diagnosis, improper treatment, and poor patient outcomes. The lack of adequate training further contributes to increased turnover rates, intensifying the burden on the healthcare system.

### Medical products and technology

We study identified minimal challenges in the availability and accessibility of essential medicines and diagnostic materials, thereby benefiting the integration of TB, DM, and TC programmes.

#### Supply of medicines and diagnostic kits

A crucial facilitator for the successful implementation of the TB programme is a smooth supply of medicines and diagnostic kits. The NTEP ensures timely and effective treatment by implementing a drug procurement and distribution system, collaborating with other agencies to ensure quality.

Yes, sufficient kits are there. For TB, we have a portal which is implemented throughout the country, that is the dispensation module. At what place what drugs are available or are over is known through that portal. Hence immediate actions can be taken.* – IDI# 2*

Participants acknowledged the current logistics supply chain system as a viable option for long-term medical supply chain management for both diseases. Contrarily, the frequent stockouts of diabetes management supplies and medicines in India hinder the implementation of diabetes management programmes. This leads to interruptions in care, suboptimal control, increased complications, and reduced quality of life. Moreover, these stockouts affect patients’ and providers’ confidence, resulting in a loss of trust in the healthcare system.

For three months there has been a shortage of medicines. This happens very frequently when it comes to DM medicines and other supplies. Few months ago, I had no supply of Metformin for two months. There is also insufficiency in the glucometer strips due to which the testing is hampered.* – IDI# 15*

Some respondents expressed a belief that DM control medicines are prohibitively expensive, resulting in costly prescriptions for patients from private retail pharmacies. Economically disadvantaged patients noted that the inability to afford out-of-pocket medicines during stockouts in public health systems lead to lower doses and poor medication adherence. Diagnosing diabetes requires more than just conducting blood glucose tests; it necessitates access to medical supplies to ensure effective management in India.

The present integrated medicine supply system, which is managed by the Pharmaceutical Fund and Supply Agency, does not adequately accommodate DM supply. We frequently run out of stock. Even at private wholesale providers, the needed commodities for DM care are not always available. Patients are compelled to purchase medications from private retail pharmacies at exorbitant prices.* – IDI#19)*

#### Ni-kshay Portal, Ni-kshay Poshan Yojana, and Ni-kshay Aushadhi Yojana

The NTEP in India has implemented three initiatives to improve TB programmes: Ni-kshay Portal, Ni-kshay Poshan Yojana, and Ni-kshay Aushadhi Yojana. Ni-kshay Portal monitors TB patients, providing real-time data on diagnosis, treatment, and outcomes. The Ni-kshay Poshan Yojana provides nutritional support and the Ni-kshay Aushadhi Yojana ensures the free supply of anti-TB drugs. These initiatives have not only enhanced the quality of TB care but also facilitated the smooth supply of medicines and diagnostic kits.

### Information

The health information system theme emphasises the significant of data-driven decision-making and highlights the necessity for a robust health information system. Such a system would play a crucial role in facilitating the integration of TB, DM, and TC programmes, as well as promoting data sharing across healthcare providers.

#### Less awareness of Health Management Information System among healthcare professionals and community

The Health Management Information System (HMIS) is a web-based monitoring system in India designed to track health programmes, policies, and interventions. It plays a crucial role in grading facilities, identifying prevent areas, and reviewing the programme implementation plan. However, a lack of knowledge about HMIS among healthcare professionals and community members results in its underutilisation and hinders data-driven decision-making. Ultimately, this has an impact on the quality and effectiveness of healthcare services.

I have noticed that many health care professionals are not familiar with the Health Management Information System and its benefits. This lack of awareness can lead to incomplete and inaccurate data collection, which can ultimately affect the quality of health care services. It is important for health care professionals to be trained and educated on the HMIS to ensure that accurate data are collected and used to improve the health care system. *– IDI# 2*

#### Presence of HMIS for every disease

The integration of TB-DM-TC relies on dedicated HMIS systems for each disease. The NTEP's Ni-kshay tracks TB patients from diagnosis to treatment completion, offering real-time data on cases, treatment outcomes, and drug resistance patterns. The NPCDCS' portal monitors cancer and diabetes patients, while the NTCP's separate portal contributes to enhance disease control and management across the country.

For NTCP, there is a separate portal where all the data are put up to the state and district level. Quarterly data are used. – *IDI# 9*

There is one portal for NCDs. The data entry requires around 10 minutes for one patient. The portal has info about HTN, DM, and about tobacco, and we get to know only if that person is suffering from TB or not.* – IDI# 19*

#### Underutilised NPCDCS portal

Our data suggests that the NPCDCS portal is outdated and underutilised, hindering the implementation of integrated programmes. With a paper-based reporting system and limited human resources, updating information on a vast array of diseases becomes challenging. This underutilisation significantly hinders efficient health management and data recording for DM.

We have different registers for different diseases and we have paper-based records of all the patients when it comes to DM. I do not know about the other two conditions.* – IDI# 14*

#### Availability of resources for information, education, and communication activities

Some participants suggested that the information, education, and communication (IEC) activities are crucial for spreading awareness and promoting health-seeking behaviour in the community. Resources such as funding, trained personnel, and appropriate tools facilitate effective implementation.

I believe that availability of resources for IEC activities is crucial for the successful implementation of any health programme. These resources can help in creating awareness. This will not only increase the utilisation of the system but also improve the quality of data being entered in HMIS. – *IDI# 1*

### Finance

Financing refers to the mechanisms used to fund healthcare services. Our findings for this block highlight the need for adequate funding for integrated programmes and emphasise the importance of innovative financing mechanisms for the sustainability of integrated care.

#### Availability of financial resources

Our respondents highlight the government's consistent budget allocation for the health sector, ensuring adequate funds for the procurement of medicines, equipment, and other resources. This commitment has led to the successful implementation of health programmes like NTEP and NPCDCS, the recruitment of additional healthcare professionals, and the facilitation of health promotion campaigns and awareness programmes, contributing to improved population health outcomes.

For TB, funds are sufficient. Occasionally, a delay is observed. For other states, as per disease burden and geographical needs, it is allocated to states/districts. Usually, funds are sufficiently provided to all states. – *IDI# 5*

#### Different financial mechanisms for each programme

Our data portray different financial mechanisms for each programme, potentially hindering the integrated implementation of health programmes. These mechanisms contribute to discrepancies in funding allocation, which in turn affect the overall quality of the health system and procurement processes. Additionally, differences in compliance requirements and timelines can result in delays and inefficiencies in implementing the integrated programme. To address these challenges, our data suggests the need for an integrated financial system to facilitate holistic resource allocation and management.

I think one of the biggest challenges we face is the separate funding mechanisms for each national health programme. It's difficult to manage the allocation of resources and ensure that each programme is adequately funded.* – IDI# 16*

#### Limitations in integrating programmes based on financial schemes

The efficient utilisation of resources in the health sector faces a substantial barrier in the form of limitations arising from integrating programmes based on financial schemes. The challenge of incorporating financial schemes within health programmes emerges from variations in funding mechanisms, funding availability, and procedural differences. This disparity can lead to an inequitable distribution of resources, leading to adverse health outcomes and influencing geographical or demographic priorities.

No, funding cannot be integrated. Activities can be integrated together but not funding. There are common cross-cutting strategies or a common budget is provided for few activities. It can be integrated for a particular activity but is not feasible for the whole programme.* – IDI# 17*

#### Involvement of the private sector

The involvement of the private sector in health financing can serve as both a facilitator and a barrier to the effective implementation of national health programmes, given the diverse opinions of participants. While private sector involvement could enhance quality and coverage, variations across programmes lead to discrepancies in funding and resource allocation. Study participants emphasised the importance of managing private sector involvement to complement the goals of the national health programme.

Participants also emphasised the importance of cross-sectoral planning and involving profit-making firms through corporate social responsibility at the city district level. This strategy has the potential to mobilise resources and funding, ultimately contributing to the improvement of community health.

Yes, it is happening in NTEP. For an increase in private notifications, we are taking help from private agencies, which is currently functional in 35 districts. Current financing is good. Paper-based needs are always there. If expenditure is not there only then there is no point. When we get saturated with our own funds then we go to the private sector.* – IDI# 11*

### Governance and leadership

Effective integrated care management demands strong governance and leadership, necessitating robust leadership, political commitment, and the identification and resolution of barriers and facilitators.

#### Underdefined systems for the involvement of private practitioners

The lack of clear frameworks and structured systems for involving private practitioners in integrated health programs hinders their effective implementation, especially in rural and remote regions of India. This ambiguity, combined with inadequate incentives and limited recognition, discourages private sector engagement and contributes to disparities in healthcare service delivery. Despite functioning independently, the absence of recognition may demotivate their participation. This deficiency can lead to the duplication of efforts and inefficiencies in healthcare service delivery.

Public private partnership (PPP) is there but has not worked out on all levels. If you want to incorporate private sectors, there are possibilities of PPP like that in TB. It can be done for DM too. There are few non-governmental organisations working with NTCP, but there are no private hospitals. That is not the domain for them. While doing PPP, there should be some mandated corporate social responsibility activities regarding the promotion of harmful effects. *– IDI# 4*

#### Well-designed programmes

The effective implementation of healthcare services relies heavily on well-designed national health programmes; for example, NTEP, NCD, and TC programmes concentrate on specific health issues, employ evidence-based strategies, and have clear goals, objectives, and structured activities. Consistent monitoring and evaluation mechanisms ensure prompt feedback and corrective measures. The study participants unanimously agreed that these programmes provide a framework for the coordination and integration of healthcare services, ensuring timely and efficient delivery.

I believe that the current national health programmes are well designed and have the potential to make a significant impact on the health of the population. The programmes have been developed based on extensive research and consultation with experts in the field.* – IDI# 16*

#### History of tobacco use documented in the TB and DM programme

Identifying individuals at risk of developing diseases like TB and DM relies on the assessment of their tobacco use history. These data play a pivotal role in shaping targeted prevention and control interventions and monitoring their success.

I think documenting the history of tobacco use in TB and DM programmes is a really important step in addressing the burden of tobacco-related diseases in India. By identifying patients with a history of tobacco use, we can provide them with targeted interventions and support to quit smoking or chewing tobacco. – *IDI# 9*

#### Lack of optimum coordination, communication and discussion

Our data suggest that the effectiveness of newly integrated health programmes depend on deliberate and structured collaboration among existing national health programmes. While integration aims to unify service delivery, such efforts often face operational silos. Facilitated by open communication and discussions, this collaborative approach helps identify areas of overlap, prevents duplication, highlights mutual benefits, and addresses challenges or barriers that could impede the programme’s success. Very importantly, the cross-talk between all three programmes for integration was emphasised in the data from sites in both states.

While the new integrated programme may face challenges in regulation and accountability for policymakers and programme managers, strong regulation and collaborative technical standards will likely attract interest from implementers and funding agencies. Most participants also agreed that establishing a TB-DM-TC integrated programme within the healthcare delivery system is feasible, provided the DM programme’s gaps are addressed and specific tasks are identified for all levels.

I believe that optimum communication and discussion are essential to ensure equal benefit for the participating programmes. As a health care professional, I have seen first-hand benefits of collaboration and coordination between different programmes, especially when it comes to delivering health care services in remote and underserved areas. Effective communication ensures that all stakeholders are aware of the programmes and their objectives, reducing the duplication of efforts and ensuring that resources are utilised efficiently.* – IDI# 22*

## DISCUSSION

Our primary goal in this qualitative study was to investigate potential health system barriers and facilitators that merit consideration in the integration of the TB-DM-TC programme. Below we explore the implications of these findings for programme coordinators and implementers in India, while offering insights into potential strategies for overcoming these challenges and optimising the integration process.

The absence of collaboration between TB-DM and TB-TC integrated services primarily stems from the lack of clear operational guidelines. In contrast, the TB-HIV integration program demonstrated notable success, which can serve as a valuable model to guide the integration of TB, DM, and TC initiatives. The effectiveness of such integration relies on developing services that are comprehensive, continuous, competent, compassionate, and cost-effective, all planned within the limitations of available resources [11]. For illustration, patients with elevated blood glucose levels are typically referred to NCD clinics. However, not all individuals with DM undergo TB testing, as only those with symptoms are screened and directed to TB clinics. This approach may not be comprehensive and could result in missed TB cases in DM patients [12]. Considering the high prevalence of DM in the community and the modes of diagnosis and treatment, testing all patients with DM for TB may seem far-fetched, as such a strategy could potentially burden the health system with unnecessary TB testing. To address this challenge, a well-defined, evidence-based approach to TB screening that considers the prevalence of TB and DM in the community and available resources is necessary for all DM patients.

The prevailing referral system has proven inadequate in providing feedback on referral outcomes, leading to a lack of communication and follow-up between primary care facilities and referral hospitals. This deficiency reflects a misalignment with current standards, leading to patients falling through the cracks of the system despite substantial efforts, and underscores the urgent need for a robust and stringent monitoring system to ensure that all patients registered within the public health framework are consistently tracked until recovery. Therefore, establishing efficient communication channels and robust referral systems becomes imperative. We also found that self-referrals to doctors and higher specialisation levels hinder healthcare delivery in rural areas. This fragmentation of health services can lead to poorer outcomes. Improving patient education and awareness about the referral system hierarchy is crucial for coordinated care. Strengthening the referral system thus requires effective communication, coordination, and monitoring mechanisms to ensure compliance with the hierarchy.

Based on our findings, we highlight that the scarcity of TC centres hinders the integration of smoking control services into the TB-DM programme. To improve access, the establishment of more centres, pilot tests, and the conduct of pre-implementation testing in real-world environments are recommended. Context-specific training materials should also be developed for healthcare personnel to address issues such as stigma, ignorance, low knowledge, and illiteracy [13]. Tobacco cessation counselling training initiatives have progressed slowly, indicating the need for continued efforts to strengthen NTCP implementation and enhance the capacity of healthcare professionals. Additionally, the government could explore offering incentives to private healthcare providers for their involvement in TC programmes to encourage their active participation.

We identified potential opportunities within the Indian health system to implement the proposed TB-DM-TC strategy, which aligns with prior studies. Notably, the well-organised TB control programme [14] and the partially institutionalised TB-DM screening [8,15] serve as foundations for potential integration. This finding underscores the existence of a robust framework upon which to integrate DM and TC programmes. The well-established and widely recognised system in the Indian health system provides an opportunity to leverage existing infrastructure and expand the scope of care to include DM and TC services.

Despite established regulations and standards, service delivery for TB/DM comorbidity remains deficient. This is a matter that cannot be neglected, particularly as India strives to achieve TB-free status five years ahead of global targets. Vigilance within healthcare systems is paramount, and the community must be informed about this critical issue.

In line with other studies [1,7,8], we identified the main barriers to integrated care: inadequate knowledge among healthcare providers, a lack of diagnostic tools and equipment (especially DM kits and medicine supplies), and insufficient attention to DM control and smoking control programmes. The lack of knowledge on HMIS contributes to these barriers, impacting the motivation and engagement of healthcare workers and community members in utilising the system. Healthcare workers may perceive HMIS as an additional burden to their already demanding workload, leading to resistance in its implementation. Therefore, effective communication strategies are critically needed. Moreover, the community's underappreciation of TB/DM comorbidity leads to a lack of awareness and knowledge about tobacco and alcohol use. Addressing this issue requires community-based interventions that aim to create awareness, reduce stigma, and motivate individuals to seek healthcare services. Additionally, health workers should be educated on the importance of intervening in smoking for TB patients, and providers must receive proper training [13].

We also highlight the importance of cross-programme and cross-sectoral collaboration for efficient execution and sustainability in integrated programmes. This collaborative approach not only promotes resource efficiency, but also minimises duplication and ensures the continuity of care. The significance of multisectoral coordination and cooperation, particularly involving private primary care providers in the collaborative management of TB-DM, has been discussed in an Indonesia-based study [16] and consistently advocated by other research [1,17].

The establishment of culture-sensitive infrastructure is crucial for the success of preventive operations, such as screening, to ensure user engagement and early acceptance. The suggestion is for tri-directional screening of TB-DM-smoking at community outreach and primary healthcare facilities, along with intensive health promotion activities to increase community health literacy [7]. However, if the IEC messages are not culturally appropriate or not translated into local languages, they may not effectively reach the target population. Hence, modifying or adapting the IEC materials to the local context is necessary. Additionally, enhancing the national health information system is recommended to address reporting and surveillance issues [1,17]. Implementers and decision-makers should collaborate to create an integrated health information system, establish shared objectives, and provide training for care providers on reporting and recording. The implementers can provide the decision-makers implementation tactics for tailored interventions within the resource-constrained Indian health system.

### Strengths and limitations

The strengths of our study include the use of qualitative data collection methods that enabled an in-depth exploration of the participants' experiences and perspectives, providing rich and nuanced data. The inclusion of healthcare providers from all strata in the study also provided a well-rounded perspective on the challenges faced and potential solutions. Finally, the use of multiple data sources for triangulation purposes ensured the reliability and validity of the results.

This study's limitations include its narrow geographic scope, as it was conducted in two districts across two Indian states, making the results context-specific and not applicable nationwide. We also only engaged a limited number of NTCP stakeholders, which would have led to a less thorough understanding of tobacco-related barriers and programme framework facilitators.

### Recommendations

The participants of our study recommended establishing an independent integrated TB-DM-TC programme using existing resources, developing joint technical guidelines for standardisation, and planning for financial resources and national budgeting. They also advocated for integrating patient record software onto a single platform with a unique identification number. Furthermore, they also noted a need for enabling single visits for TB patients with comorbidities, establishing communication across all three programmes, and involving private practitioners in screening and sensitising them for Ni-kshay registration. There must be a rigorous implementation of the feedback and referral system, with training modules that incorporate joint planning, implementation, monitoring, and evaluation. Additionally, there should be provisional psychosocial support for patients, and the use of digital health technologies should be enhanced among communities. These recommendations aim to improve patient outcomes and efficiency in national health programmes and are accompanied by their corresponding action points or benefits (**Table 1**).

**Table 1:** Recommendations by themes with their action points

	Action points/benefits
**Service delivery**	
Deployment of rigorous screening procedures	Rigorous screening procedures for DM during TB treatment are essential in identifying patients with comorbidities by creating standardised protocols.
Single visit for patients with comorbidities	The implementation of a single visit for TB patients with comorbidities will improve patient experience and reduce the burden of multiple visits.
Single window for service provision	The provision of a single window for service provision will enable effective communication and coordination among the three programmes, leading to improved patient outcomes.
Rigorous implementation of the linkage mechanisms	The rigorous implementation of the feedback and referral system will enable timely and effective communication among healthcare personnel, improving patient outcomes and programme effectiveness.
Provision of psychosocial support to patients	Develop a coordinated approach for the provision of psychosocial support to patients with TB, DM, and other conditions, addressing their mental and emotional health needs.
**Health workforce**	
Incorporating joint training modules	Training modules incorporating joint planning, implementation, monitoring, and evaluation will improve healthcare personnel's skills and knowledge, leading to effective programme implementation.
Address overburden through adequate staffing	Mitigate the work pressure by making sure there is a sufficient and evenly distributed workforce to handle the needs of the integrated programme.
Encourage the enthusiasm and dedication among health workers	This can be accomplished by giving them opportunities for professional progress within the integrated programme, as well as recognition and support.
**Medical products and technology**	
Address the cost-effectiveness and stock-outs of diabetes related medications and supplies	Investigating tactics like subsidies, bulk procurement, or alliances with pharmaceutical firms may be beneficial.
**Information**	
Integrated software on a single platform for seamless data sharing	Integrating the patient record software onto a single platform with a unique identification number can significantly improve the delivery of healthcare services.
Sensitise health professionals to the HMIS	Campaigns for focussed training and information dissemination can help accomplish this.
**Finance**	
Involve Private Practitioners in screening	Involving private practitioners in the screening of TB and DM and sensitising them for Ni-kshay registration will increase the reach of national health programmes, especially in remote and rural areas.
Utilise integrated financing strategies to prevent duplication	Establishing a single budget may benefit from doing a thorough financial assessment by identifying overlapping expenses.
**Governance and leadership**	
Improve stakeholder collaboration, communication, and coordination	Extensive stakeholder mapping, the establishment of a coordinating body to monitor integration efforts, the implementation of standardised reporting practices, and the adoption of a conflict resolution protocol are some of the actions that can be taken to achieve these goals.

DM – diabetes mellitus, HMIS – Health Management Information System, TB – tuberculosis

## CONCLUSIONS

Here we outlined the barriers and facilitators to implementing an integrated TB, DM, and TC programme within India's public health systems. The findings underscore the urgent need for a comprehensive and cohesive approach to tackle India's burden of TB, DM and tobacco smoking. Furthermore, it highlights the importance of establishing operational guidelines to foster collaboration among various public health initiatives, setting a precedent for integrating additional programmes within India.

Our findings suggest that leveraging the established NTEP infrastructure could serve as a robust foundation for incorporating DM and TB services within primary care, underscoring the need to address deficiencies in service delivery for TB/DM comorbidity, especially in light of India's aspirations to achieve TB-free status. The findings of our study also strongly advocate for the convergence of the three programmes into a single-window approach, along with the establishment of effective feedback and referral systems, and the development of harmonised digital data entry platforms. A multifaceted approach is recommended for policymakers and healthcare system management.

Our findings support the implementation of culturally sensitive infrastructure, community-based interventions, and health promotion activities to enhance user engagement and facilitate early detection. They also underscores the importance of strengthening the national health information system and adopting targeted interventions within resource-constrained environments to support successful integration efforts. Furthermore, they highlight the need for ongoing research to continually evaluate the efficacy of integrated models in addressing complex health issues in the Indian context.

## Additional material


Online Supplementary Document
