# Supplementary material for: Exploring potential barriers and facilitators to integrate tuberculosis, diabetes mellitus, and tobacco control programmes in India
Source: J Glob Health. 2025 Aug 15;15:04230. doi: 10.7189/jogh.15.04230 (PMC12355441; doi:10.7189/jogh.15.04230)
Supplement: Online Supplementary Document [file jogh-15-04230-s001.pdf]

Supplement to: Mutalikdesai N, Tonde K, Shinde K, Kumar R, Gupta S, Dayma G, Krishnan A, Juvekar S, Santosa A, Ng N, Patil R. Exploring potential barriers and facilitators to integrate tuberculosis, diabetes mellitus, and tobacco control programmes in India. J Glob Health. 2025;15:04230.

Key-Informant Interview in India and Indonesia

Objectives
1. To understand the existing programs in relation to TB, DM, and smoking control programmes
2. To explore the facilitators and barriers for the integration of the three programmes at the national, provincial/state and district levels

Framework
The key information interview is developed based on the six building blocks of the health system as the main framework, which includes the following dimensions: (1) Service delivery, (2) Health workforce; (3) Health information system; (4) Financing; (5) Governance and leadership; and (6) Access to essential medicine.

Interview Guide

Opening questions:
1. Which institution are you working at?
2. What is your current position and responsibility?
3. How are the states (province/district/city) dealing with the dual burden of communicable and non-communicable diseases?
4. Has the PIP (Programme Implementation Plan) been tailored to the disease burden in the state and district level?
5. Is there a budget allocation?
6. Are you doing any implementation research in this regard?

Please indicate how often have your institution been working together with the following institutions.

Institutions	Never	Rarely (Every other year)	Sometime (1-3 times a year)	Often (4-5 times per year)	Very often (At least 6 times per year)
A.					
B.					
C.					
D.					
E.					
F.					

Please list all other institutions **apart from the ones mentioned above** that your institution has been collaborating with.

Interview questions related to the health system block:

Service delivery

- How is the bi-directional screening of TB and DM organised at different levels of the health system? Is it working well? Does it require more strengthening? What do you think will help enhance integration?
- What are the opportunities that you see and the barriers they face when they plan to do the TB and DM screening?
- How do we ensure the comprehensive screening of TB and DM through both active and passive screening?
- What is the current situation regarding the above and what are suggestions for more comprehensive integration of communicable and non-communicable disease programme?

Health workforce

- The human resources: Do they have the personnel to do the screening/disease management? Sufficiency? Task shifting?
- Training: do you have the specific knowledge to do the integrated disease control program (e.g. TB-DM)? Have you been trained in conducting screening and disease control?
- Coordination between the different disease program staff

Health information system

- Current disease program information system: the linkages of infrastructure in the different programme, the load of recording and reporting
- The current plan of an integrated information system
- Are the data used in the coordination meeting?
- Are they using the data for evidence-based decision making?

Financing

- Fund-flow mechanisms - do the fund come to the district/PHC level?
- Are government funding available for TB/DM/Tobacco control programme? Assess differences between the states/districts
- Would you think the integration of Communicable and Non-Communicable program is feasible based on the financing scheme? Why?
- Is the private sector involved in financing the integrated programs? Continuity of the funding?

Governance and Leadership

- What kind of inter-departmental and inter-sectoral cooperations/coalitions they have within and outside the government?
- Are there guidelines? Have they been disseminated? What do you think about the guideline?
- Leadership and coordination for sharing resources (diagonal sharing) - whether the rich resources would like co-share with another program
- Fund flow mechanism
- What are the common challenges you would face in the integration of the programme?
- Is there any policy to involve private sectors and NGOs in the integration of communicable and non-communicable diseases control programme.

Access to essential medicine

- Do you have sufficient screening and diagnosis kits (sputum test, GeneXpert, etc) for tuberculosis and diabetes?
- Do you have sufficient essential medicine for treatment for tuberculosis and diabetes?
- What are the estimated costs of treating a patient with TB and/or DM?

Please think of all the actions that **your institution** can do to facilitate the integration of TB, DM and tobacco programme. There is no right and wrong answer, please list all the actions that you can think of.

Please think of all the actions that **other institutions mentioned above** can do to facilitate the integration of TB, DM and tobacco programme. There is no right and wrong answer, please list all the actions that you can think of.

